# AlGaN HEMT Structures Grown on Miscut Si(111) Wafers

**DOI:** 10.3390/ma16124265

**Published:** 2023-06-08

**Authors:** Alexei V. Sakharov, Dmitri S. Arteev, Evgenii E. Zavarin, Andrey E. Nikolaev, Wsevolod V. Lundin, Nikita D. Prasolov, Maria A. Yagovkina, Andrey F. Tsatsulnikov, Sergey D. Fedotov, Evgenii M. Sokolov, Vladimir N. Statsenko

**Affiliations:** 1 Submicron Heterostructures for Microelectronics, Research and Engineering Center, RAS, 26 Politekhnicheskaya, 194021 Saint-Petersburg, Russia; 2 Ioffe Institute, 26 Politekhnicheskaya, 194021 Saint-Petersburg, Russia; 3 Epiel Joint Stock Company, 6/2 Akademika Valieva, 124460 Zelenograd, Russia; 4 ONSI Ltd., 6/2 Akademika Valieva, 124460 Zelenograd, Russia

**Keywords:** HEMT, carrier mobility, AlGaN, Si, MOVPE, miscut

## Abstract

A complex study was performed on a set of AlGaN/GaN high-electron-mobility transistor structures grown by metalorganic vapor phase epitaxy on miscut Si(111) wafers with a highly resistive epitaxial Si layer to investigate the influence of substrate miscut on their properties. The results showed that wafer misorientation had an influence on the strain evolution during the growth and surface morphology, and could have a strong impact on the mobility of 2D electron gas, with a weak optimum at 0.5° miscut angle. A numerical analysis revealed that the interface roughness was a main parameter responsible for the variation in electron mobility.

## 1. Introduction

III-V-based high-electron-mobility transistors (HEMTs) have become a part of modern electronics, actively replacing existing Si devices. GaN has a unique combination of properties such as a large band gap (~3.4 eV, three times that of silicon), high-electron-saturation velocity (~2.5 × 10^7^ m/s) [1], a high-breakdown electric field >3 MV/cm [2] and electron mobility up to ~2500 cm^2^/V·s at room temperature [3] for 2D electron gas formed at the Al(Ga)N/GaN heterointerface. Such structures also show high radiation resistance and good thermal stability in harsh environments [4]. Remarkable progress in the growth and processing of III-N HEMTs results in high-power [5] and high-frequency [6] devices. A strong piezoelectric effect and a large spontaneous polarization allow for the obtaining of high two-dimensional electron gas (2DEG) concentration at the interfaces, even without intentional doping; therefore, conventional HEMTs based on III-nitrides are inherently normally on. However, fabrication of normally off GaN HEMTs are also possible [7].

GaN-based structures can be grown on a variety of substrates, ranging from GaN and AlN to sapphire, SiC, silicon and other less common ones. In spite of great progress, GaN and AlN wafers are still too expensive and limited in size; sapphire is cheaper but has a low thermal conductivity. Synthetic diamond, with its outstanding thermal conductivity and breakdown field, could be the best choice for high-power devices, but such wafers are limited in size as well, and fostering epitaxial growth on them is very challenging [8,9]. SiC and silicon are the remaining substrate options. The cost-saving effect of increased wafer size encourages the use of larger and larger wafers, and silicon wafers are the only ones widely commercially available at 200–300 mm size. Additionally, while 200 mm SiC is on the way [10], it is expected to be quite expensive. GaN HEMT grown on 300 mm Si wafers was recently demonstrated [11].

There are several approaches that can be used to address the problem of significant thermal and lattice mismatch between GaN and Si. One approach involves introducing thin, usually low-temperature AlN interlayers [12] or implementing an Al(Ga)N/GaN superlattice structure [13]. Another method implies the use of a step-graded or gradient AlGaN buffer [14]. The basic concept behind both methods is to introduce compressive stress to compensate for the tensile stress that arises upon cooling. The first approach is more versatile, and can potentially provide an almost unlimited thickness of the structure, while the second one seems slightly easier from a technological standpoint.

It is well known that substrate miscut severely affects the growth mechanism and properties of the grown layers. While miscut wafers have become the de-facto standard for light-emitting diodes grown on sapphire, the situation is not as clear for SiC and Si. There are some publications addressing the influence of wafer miscut on growth on SiC [15,16], sapphire [17,18] and Si [19], but there are limited data on HEMT structures grown on miscut Si(111) wafers. The paper [20] reported a gradual improvement in HEMT properties with the miscut angle increased to 1.0 degree, but no analysis was presented. On the other hand, interface roughness scattering (IFR) is known to be one of the scattering mechanisms severely limiting the mobility of 2DEG of AlGaN- and InAlN-based HEMTs, especially at low temperatures [21,22]. Additionally, since wafer miscut angle may affect the morphology of the layers grown, selecting an appropriate substrate could be beneficial in terms of obtaining better-performing devices.

In this work, we studied the influence of Si(111) wafer miscut on the growth and properties of AlGaN-based HEMT structures.

## 2. Experimental

The structures were grown by metalorganic vapor phase epitaxy (MOVPE) using our in-house Dragon-125 epitaxial system specially designed for III-N growth. It has a horizontal flow reactor with an inductively heated susceptor with 3 × 2″ or 1 × 100 mm wafer capacity and is equipped with a two-wavelength (638 nm and 405 nm) multi-point laser reflectometry/deflectometry system (also in-house developed), that allows precise in situ measurements of growth rate and wafer bowing, which is crucial for growth on silicon substrates. A properly designed reactor enables the epitaxial growth of III-N layers with high growth rates due to reduced parasitic reactions [23,24,25]. Trimethylgallium, trimethylaluminum and ammonia were used as precursors. Hydrogen, nitrogen and their mixture served as carrier gases. Ferrocene and propane were used as dopants to grow insulating buffer layers.

It is well known that conductive or low-resistivity wafers can degrade HEMT performance at high frequencies due to the introduction of additional RF losses [26], high-resistivity (HR) float zone (FZ) Si wafers are generally preferred. However, the high growth temperatures required to grow high-quality layers can cause thermally induced stress in the wafer during the growth process and subsequent cooling, which can lead to plastic relaxation of Si. Plastic deformation was reported in a paper [27] on the growth of III-N on Czochralski (CZ) and FZ silicon substrates. We observed the same effect for some FZ wafers as well. Differences in mechanical properties of CZ and FZ silicon [28] as well as their temperature dependencies [29] were extensively studied earlier. All three of the aforementioned papers reported that CZ substrates were more deformation-resistant than FZ ones. Moreover, p-type Si has higher upper yield stress than undoped or n-doped [30]. Last but not least, CZ wafers are more commercially available. To address these challenges, Epiel JSC developed a good alternative to the HR FZ substrates standard for RF HEMT epitaxy: special “HR-Si-on-CZ-Si” templates, consisting of a ~110 μm high-resistivity (>2000 Ω·cm) Si layer epitaxially grown on a standard commercial 575 μm-thick boron-doped CZ Si substrate by chloride vapor phase epitaxy [31,32]. These templates provide good mechanical properties with low RF losses and allow the conductive part of a substrate to be removed during the thinning procedure after growth of the whole structure if necessary. A conductive part of a substrate can be removed during the thinning procedure after the growth of the whole structure if necessary.

The process began with an annealing of the template in hydrogen atmosphere to remove a native oxide from the surface. Then, a ~200 nm AlN layer was deposited at 1100 °C with V/III ratio of ~800 to prevent Si wafer etching by gallium (the so-called meltback etching effect [33]), so “AlN-on-Si” templates were prepared. After that, in another process, a six-layer step-graded AlGaN buffer ending with a GaN layer (hereinafter referred to as ‘LT-GaN’) with total thickness of ~3.1 μm was grown at 1050 °C to compensate for the strain arising during the cooling down of the epistructure due to the difference in thermal expansion coefficients between Si and III-N materials. The step-graded AlGaN buffer not only introduces necessary strain but also effectively reduces dislocation density [34]. Fe and C co-doping was used in buffer layers since it results in higher breakdown voltages and provides a wider growth parameter window [35].

**Figure 1:**
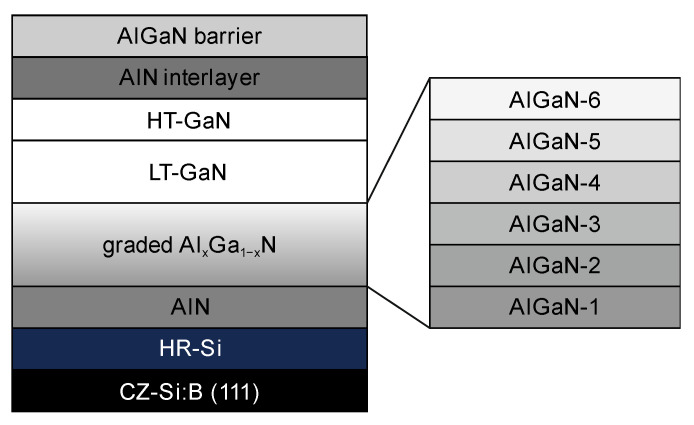
Schematic of the epitaxial structures.

A ~400 nm undoped GaN channel layer (hereinafter referred to as ‘HT-GaN’) was grown at a higher temperature of 1100 °C to reduce unintentional carbon incorporation and improve carrier mobility [36]. The V/III ratio was gradually increased from ~1000 for high-Al AlGaN layers to 2000 for low-Al AlGaN layers; both GaN layers were grown with a ~2500 V/III ratio. Afterward, the temperature was lowered back to 1050 °C, and the structure was completed with a 1 nm AlN interlayer and 26 nm Al_0.27_Ga_0.83_N barrier layer. A thin AlN interlayer almost completely eliminates the penetration of the electron wavefunction into the ternary AlGaN barrier, enabling increased electron mobility by significantly reducing alloy-disorder scattering [22,37]. The epitaxial structure is schematically shown in Figure 1. SiN capping was not used to ease the Hall effect measurements. All samples were grown under the same growth conditions; the only difference was the wafer miscut angle. 100 mm Si(111) wafers with a misorientation angle up to 2.0 degrees towards $\left[1\bar{1}0\right]$ and zero miscut to $\left[2\bar{1}1\right]$ were used. The accuracy of the miscut measurements was better than 0.05°.

A set of samples was grown on the templates, with miscut angles of 0, 0.5, 1.0 and 2.0°, referred to as M0, M5, M10 and M20, respectively. X-ray reciprocal space mapping (RSM) around the GaN and AlN (11.4) reflections was carried out, as well as measurements of X-ray diffraction (XRD) curves using a high-resolution D8 Discover Bruker AXS diffractometer equipped with a tube with a copper rotating anode, Goebel mirror, 4-bounse asymmetric monochromator (022)Ge. RSM were obtained in the geometry of the grazing reflection. The surface morphology was investigated by atomic force microscopy (AFM) using Veeco Dimension 3100 in tapping mode. The electron concentration and mobility were determined by Hall effect measurements using the van der Pauw method.

## 3. Results and Discussion

### 3.1. Curvature and Strain Evolution

**Figure 2:**
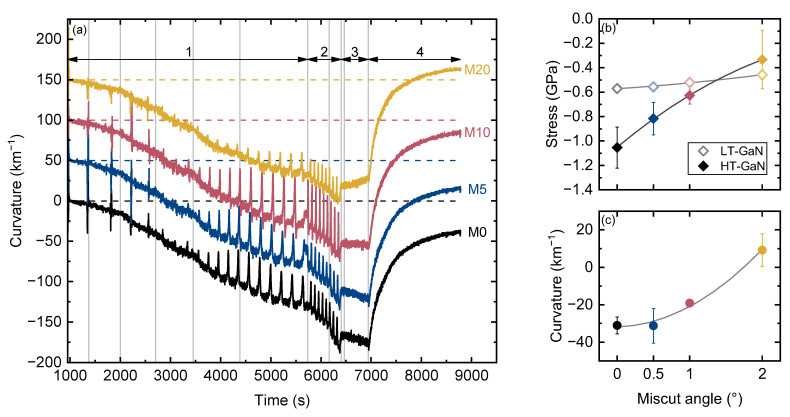
(**a**) Curvature evolution during growth on Si wafers with different miscut angle. The curves are shifted for clarity. Growth stages: 1—AlGaN step-graded buffer, 2—LT-GaN and HT-GaN layers, 3—AlN and AlGaN barriers, 4—cooling. Vertical lines delineate different layers. (**b**) Incremental stress during LT-GaN and HT-GaN growth. (**c**) Final curvature of heterostructures after cooling down. Lines in (**b**) and (**c**) are guides to the eye.

Curvature evolution during the growth of the structures is shown in Figure 2. Results are presented for one structure of each type only, since they show good reproducibility. Negative curvature values indicate a convex shape, while positive values indicate a concave one. The spikes are measurement artifacts caused by the low intensity of the reflected laser beams at certain layer thicknesses due to the Fabry–Perot effect, at which the system cannot correctly determine the deflection of the beam. The four main stages of epitaxy, namely, (1) step-graded AlGaN buffer, (2) GaN layers, (3) AlN/AlGaN barrier growth and (4) cooling, are shown by arrows. Vertical lines delineate different layers. The measured thicknesses of the AlGaN layers in the step-graded buffer were about 145, 212, 275, 410, 600 and 790 nm, and the thicknesses of LT-GaN and HT-GaN were 780 and 390 nm, respectively, with practically no differences between the samples.

During the growth of AlN and high-Al AlGaN layers, practically no difference was observed between the samples, but for the last low-Al AlGaN and both GaN layers, it became prominent. To quantify this difference, we estimated incremental stresses using Stoney’s equation in differential form [38]:

(1) $$\sigma_{f}=\frac{M_{s}h_{s}^{2}}{6}\frac{\partial \kappa }{\partial h_{f}}$$

where, $M_{s}$ = 229 GPa and $h_{s}$ = 685 μm are the elastic modulus and the thickness of the substrate. To determine incremental stress during LT-GaN and HT-GaN growth, we used a linear fit or curvature versus time with a known growth rate. Figure 2b shows the obtained average values of incremental stress. We found that strain relaxation depends on wafer miscut angle, although the mechanism behind this phenomenon remains unclear. We can suppose that the so-called Nagai tilt [39] reported for III-N on Si as well [40,41] may be responsible for that. Moreover, LT-GaN and HT-GaN are in different strain states. We also observed a different curvature development during HEMT barrier growth, ranging from negative for M0 to zero of slightly positive for M20; the origin of such behavior is not clear as well. The final curvature of the structures after cooling down differs significantly and is −31 km^−1^ for M0 and +9 km^−1^ for M20. Nevertheless, all of the grown samples were crack-free (except for the thin ~5 mm edge regions) with a specular surface.

**Figure 3:**
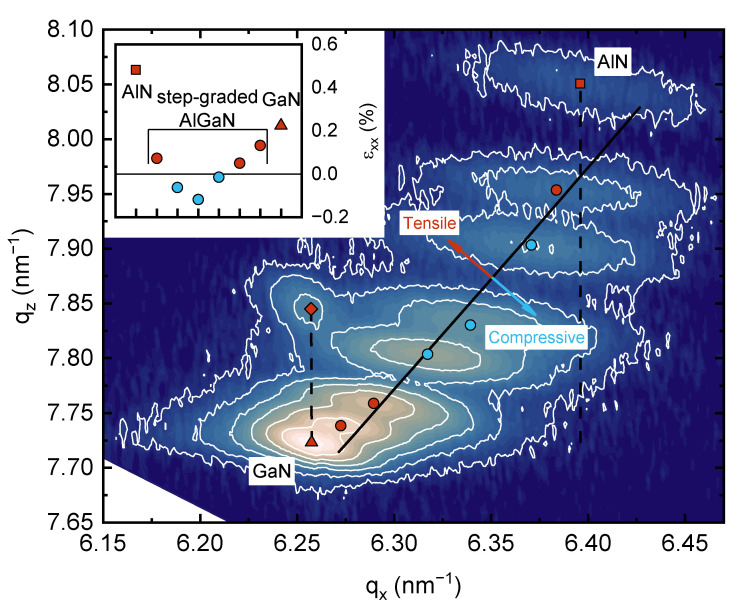
Reciprocal space map for (11.4) reflection for M0 structure grown on Si wafer with zero miscut angle. Red symbols correspond to tensely strained layers; blue symbols correspond to compressively strained layers (square—AlN; circles—step-graded AlGaN buffer layers; triangle—GaN; diamond—AlGaN barrier). The inset shows the determined strain in each layer. The black solid line corresponds to strain-free state. The dashed lines correspond to fully pseudomorphic growth with respect to AlN or GaN layer.

Sample M0 was studied by X-ray diffractometry. As a 2θ/ω scan recorded for multilayer AlGaN structure cannot provide reliable information on strain and composition, reciprocal space mapping (RSM) measurements were taken around the (11.4) reflection, which are shown in Figure 3. The black solid line represents strain-free AlGaN and the dashed lines correspond to fully pseudomorphic growth with respect to either the AlN or GaN layer. The Al mole fraction x can be determined using the following equations:

(2) $$\varepsilon_{\mathrm{zz}}\left(z\right)=-2\frac{C_{13}\left(x\right)}{C_{33}\left(x\right)}\varepsilon_{\mathrm{xx}}\left(x\right)\;\varepsilon_{\mathrm{xx}}\left(x\right)=\frac{a_{m}-a_{0}\left(x\right)}{a_{0}\left(x\right)}\;\varepsilon_{\mathrm{zz}}\left(x\right)=\frac{c_{m}-c_{0}\left(x\right)\;}{c_{0}\left(x\right)}$$

where, $a_{m}$ and $c_{m}$ are the measured lattice constants, $a_{0}\left(x\right)$ and $c_{0}\left(x\right)$ are the strain-free lattice constants and $C_{13}\left(x\right)$ and $C_{33}\left(x\right)$ are the stiffness constants for a given x. The values of the required constants for AlN and GaN were taken from [42]. The values for Al_x_Ga_1−x_N were obtained using Vegard’s law.

Symbols mark the centers of spots corresponding to different layers. The determined Al mole fractions in the layers of the step-graded buffer (from bottom to top) are 76, 62, 40, 29, 14 and 6%. The spots corresponding to Al-containing layer have an elongated elliptical shape. Upon close examination, the shape of the GaN spot appears to consist of two overlapping ellipses, which suggests that the GaN consists of layers of different stresses. This finding correlates well with the in situ curvature measurements. The strain determined for each layer is shown in the inset in Figure 3. As shown, most of the layers are tensely strained, supposedly mainly due to the differences in the coefficients of thermal expansion. However, three intermediate AlGaN layers are compressively strained. The upper barrier AlN/AlGaN layer is formed completely coherent to the underlying GaN layer, indicating pseudomorphic growth. This is in contrast to [43,44], where similar structures grown on Si and sapphire without surface passivation resulted in significantly higher strain relaxation of the barrier layer due to cracking, compared to structures passivated with a thin Si_3_N_4_ layer.

### 3.2. Surface Roughness and Mobility

**Figure 4:**
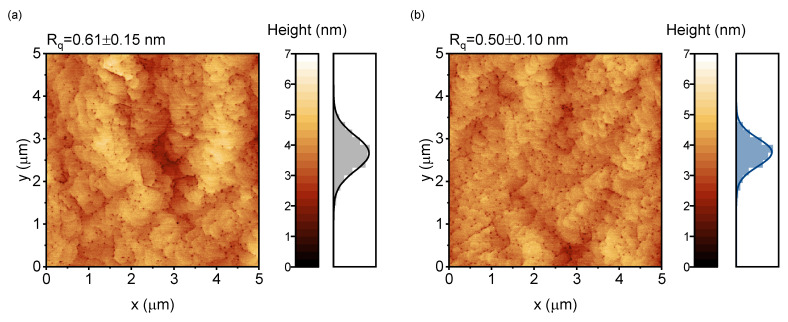

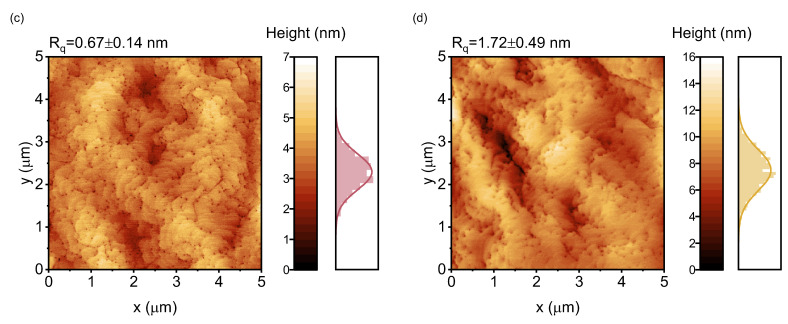
Atomic force microscopy images of samples A0 (**a**), A5 (**b**), A10 (**c**) and A20 (**d**).

Four samples referred as A0, A5, A10 and A20 were grown for AFM measurements. The samples are identical to samples M0, M5, M10 and M20, respectively, with the only difference being the absence of the upper AlN/AlGaN barrier layers. The obtained 5 × 5 μm AFM images and height distributions are shown in Figure 4. R_q_ shows the values of the root mean square (RMS) roughness averaged over the RMS roughness values of individual horizontal line profiles. As shown, sample A5, grown on the substrate with 0.5° miscut, exhibited the smoothest surface, with R_q_ = 0.50 nm, while sample A20 had a very rough surface, with R_q_ = 1.72 nm. Typically, dark spots observed in AFM images correspond to threading dislocations (TDs), so their density can be determined. The estimated TD densities in samples A0, A5 and A10 were almost the same, at 3.6–3.7 × 10^9^ cm^−2^. According to transmission electron microscopy measurements, the density of dislocations in the superficial layers of sample M0 was ~3.5 × 10^9^ cm^−2^, being in good agreement with AFM data. In contrast, sample A20 had a slightly lower estimated TD density of ~2.55 × 10^9^ cm^−2^. The trend of decreasing dislocation density with increasing sapphire substrate miscut angle was reported for AlN [45] and GaN [46].

**Figure 5:**
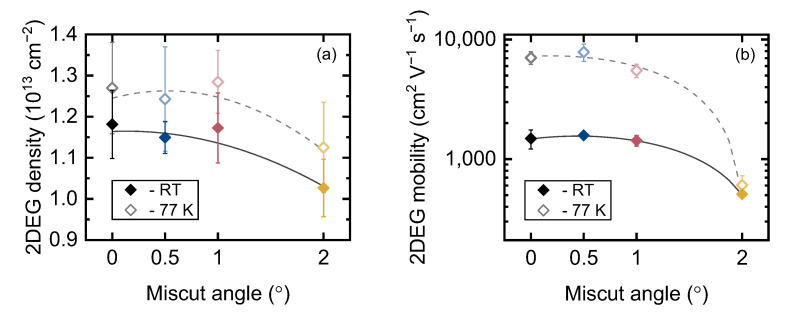
The 2DEG Hall density (**a**) and mobility (**b**) in samples grown on Si wafers with different miscut angles. Lines are guides to the eye.

The Hall density and mobility of the 2DEG in samples M0, M5, M10 and M20 were measured at room temperature (RT) and 77 K. Figure 5 shows the results, and Table 1 summarizes all the measured parameters. Again, we observed only a slight difference between the samples grown on wafers with miscut angle ≤1°, but a dramatic degradation of both concentration and mobility was observed for sample M20. Sample M5 grown on the substrate with a miscut angle of 0.5° exhibited the highest mobility at both RT and 77 K. These findings are consistent with the results for the similar structures grown on vicinal (0001) sapphire [18] and bulk GaN [47] substrates.

It is well known that interface roughness induces potential fluctuations that scatter electrons, resulting in reduced mobility. To gain a better quantitative understanding of the observed dependencies, we employed computer simulations involving numerical self-consistent solving of 1D Poisson and Schrödinger equations, along with calculations of low-field 2DEG mobility within the momentum relaxation time approximation. We considered a fully relaxed GaN channel layer with coherently strained AlN/AlGaN barrier layers. The interface roughness (IFR) scattering rate was calculated as [48]:

(3) $$\frac{1}{\tau_{\mathrm{IFR}}\left(E\right)}=\frac{e^{2}F_{\mathrm{eff}}^{2}m^{*}}{2\pi \hbar^{3}}\int_{0}^{2\pi }d\theta (1-\cos\theta )\frac{\pi \Delta^{2}L^{2}}{{\left[1+\left(q^{2}L^{2}/2\right)\right]}^{3/2}}\times {\left[\frac{\Gamma \left(q\right)}{\varepsilon \left(q\right)}\right]}^{2}$$

where, Δ and L are the RMS roughness and correlation length of the corresponding exponential autocovariance function, $F_{\mathrm{eff}}$ is the effective electric field. Since the dielectric constants of AlGaN and GaN are fairly close, the correction for image potential was neglected, i.e., $\Gamma \left(q\right)\sim 1$. All other symbols have their conventional meanings. A detailed description of the models used can be found in our previous works [49,50] and references therein. To keep the process simple, a single parameter set (including L, which served as a fitting parameter) was used, and only an RMS roughness parameter was varied during the calculation of interface roughness scattering rate, so $\tau_{\mathrm{IFR}}{\left(E\right)}^{-1}\propto \Delta^{2}$.

**Figure 6:**
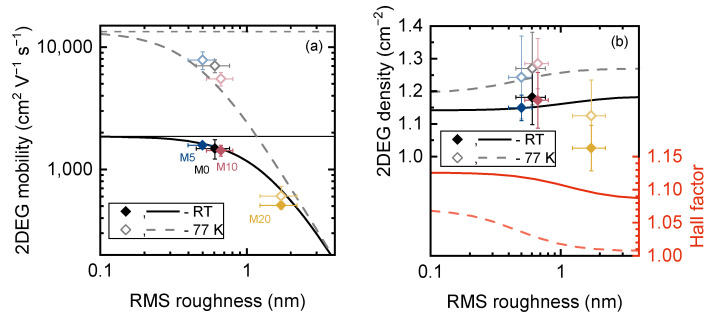
The dependence of the 2DEG Hall mobility (**a**), density and Hall factor (**b**) on the interface roughness at room temperature and 77 K. The lines and symbols correspond to calculated and experimental data, respectively.

The calculated 2DEG mobilities and concentrations were corrected by the Hall factor, which was evaluated as $r_{H}=\langle \tau^{2}\rangle ⁄\tau^{2}\;$ to ensure proper comparison with the measured data. The obtained dependencies of the 2DEG Hall mobility at RT and 77 K on RMS roughness are shown in Figure 6a as thick solid and dashed lines. Thin horizontal lines correspond to the mobility calculated without IFR taken into account. Despite the fact that not only RMS roughness, but other parameters (e.g., correlation length, dislocation density, ionized background impurity, etc.) may differ between structures, a fairly close agreement between the experimental data and the simulation results was achieved. Furthermore, varying the aforementioned parameters has a much smaller effect than varying RMS roughness, confirming the assumption of IFR scattering being a crucial factor affecting mobility.

The calculated dependence of the 2DEG Hall concentration on RMS roughness, shown in Figure 6b, agrees with the measured data for M0, M5 and M10 as well, although there is a quite large uncertainty in the experimental data. The increase in RMS roughness leads to alterations in the total Hall factor due to changes in the relative contributions of IFR and other scattering mechanisms with different energy dependence of the scattering rate to the total scattering rate, ultimately affecting the 2DEG Hall concentration. Here, we emphasize that it is the Hall concentration $n_{H}=n/r_{H}$ that changes, while the conductivity concentration n remains unchanged. However, the reason for the reduced 2DEG concentration in M20 is not completely clear, but it could be due to partial strain relaxation of the barrier layer, as reported in [47] for the samples grown on bulk GaN substrates with miscut angles >1.1°. The other possible reason is a lower polarization-induced charge density at the interface due to the different strain state of the GaN channel.

## 4. Conclusions

We performed a comparative study on AlGaN/GaN HEMT structures grown on HR-Si-on-CZ-Si templates with different miscut angles by MOVPE. Our findings demonstrate that wafer misorientation has a significant impact on strain evolution during the growth and surface morphology. Atomic force microscopy measurements showed that the samples grown on 0.5° miscut Si wafers had the smoothest surface, and, as measured by the van der Pauw method, had the highest 2DEG mobility. Further numerical analysis revealed that the increased 2DEG mobility is attributed to a decrease in interface roughness scattering rate due to lower RMS roughness. Therefore, selecting substrates with appropriate miscut angles can lead to the development of structures with better properties. However, further research is needed to fully understand the mechanism behind the layer morphology dependence on miscut angle.

## Figures and Tables

**Table 1:** The parameters of the samples.

Sample	Miscut Angle	Final Curvature, km^−1^	R_q_ (5 × 5 μm), nm	TDD, 10^9^ cm^−2^	N_2D_ (RT) 10^13^ cm^−2^	μ_2D_ (RT) cm^2^ V^−1^ s^−1^	N_2D_ (77 K) 10^13^ cm^−2^	μ_2D_ (77 K) cm^2^ V^−1^ s^−1^
M0/A0	<0.05°	−31 ± 4	0.61 ± 0.15	3.68 ± 0.31	1.18 ± 0.08	1485 ± 268	1.27 ± 0.11	7042 ± 847
M5/A5	0.5°	−31 ± 9	0.50 ± 0.10	3.70 ± 0.34	1.15 ± 0.04	1580 ± 78	1.24 ± 0.13	7851 ± 1285
M10/A10	1.0°	−19 ± 2	0.67 ± 0.14	3.57 ± 0.28	1.17 ± 0.09	1430 ± 145	1.28 ± 0.08	5508 ± 696
M20/A20	2.0°	+9 ± 8	1.72 ± 0.49	2.55 ± 0.14	1.03 ± 0.07	510 ± 179	1.13 ± 0.11	606 ± 121

## Data Availability

Not applicable.
